# Caries and quality of life in portuguese adolescents: Impact of diet and behavioural risk factors

**DOI:** 10.4317/jced.54469

**Published:** 2018-03-01

**Authors:** Javier Montero, José Costa, Isabel Bica, Rocío Barrios

**Affiliations:** 1 PhD, Tenured Lecturer Professor of Prosthodontics. School of Medicine. University of Salamanca. C/ Alfonso X El Sabio s/n. 37007, Salamanca, Spain; 2 PhD, Associate Professor. Escola Superior de Saúde, Instituto Politécnico de Viseu. R. Dom João Crisóstomo Gomes de Almeida 38, Viseu, Portugal; 3 PhD, Associate Professor. Escola Superior de Saúde, Instituto Politécnico de Viseu. R. Dom João Crisóstomo Gomes de Almeida 38, Viseu, Portugal; 4 PhD, Assistant Professor. Department of Preventive Medicine and Public Health. School of Medicine. University of Granada. Avda. de la Investigación, 18016, Granada, Spain

## Abstract

**Background:**

The aim of this study was to assess the impact of diet and behavioural risk factors on caries appearance, and on oral health-related quality of life (OHRQoL) among Portuguese adolescents.

**Material and Methods:**

An epidemiological study conducted on 782 adolescents between 11-17 years, from randomly selected public schools of the 3rd cycle of basic education. All participants were asked for self-perceived general status health, about tooth-brushing habits and about the using of toothpaste with fluoride and a Food Frequency Questionnaire. The DMFT index (decayed, missing and filled teeth) was evaluated according to WHO criteria. To evaluate the OHRQoL, the 49-items Oral Health Impact Profile questionnaire (OHIP-49) was applied.

**Results:**

Consumption more than once a week of tea with sugar, milk with sugar and biscuits were significantly associated with DMFT index. Lower levels in OHRQoL was reported by students who consumed frequently (more than once a week) fast food, chocolate flakes and those who brushed their teeth once a day or less frequently instead of 2-3 times a day.

**Conclusions:**

Frequency of consumption of sweetened/fast food was a significant factor associated with caries and quality of life.

** Key words:**Oral health-related quality of life, adolescent, diet, DMFT, epidemiology.

## Introduction

Caries and periodontal disease are the main causes of oral diseases in industrialized countries. In Europe, these pathologies have been reduced in recent years but caries continue being the most common oral pathology during childhood and the main cause of tooth loss in adulthood (1). Some observational and longitudinal studies have revealed different risk factors for caries, highlighting clinical factors such as occlusal morphology, the bacterial load in commensal flora and type of saliva (2), behavioural factors such as the frequency of brushing, the use of fluoridated products and patterns of visits to the dentist (3,4) and nutritional factors such as the frequency of consumption and the consistency of cariogenic foods (5). Within these modulatory factors, those easiest to correct are the behavioural and nutritional factors, although such habits become established from a very early age (the schooling period) and may persist or become reactivated in later years (6).

The prevention of early caries should be a priority for health authorities in matters of the Public Dental Health of a country. It is necessary to quantify the associated risk factors in order to identify high-risk groups and be able to promote effective behavioural changes that will raise the efficacy of intervention programs (7).

Epidemiological studies of oral health needs and exploring risk behaviours offer a basic tool for the planning of prevention, control and intervention programs (8). The majority of these studies just use clinical measures. However, oral health-related quality of life (OHRQoL) instruments should be a supplement to these assessments (9). Specially, the integration of OHRQoL measures in a comprehensive assessment of oral health is of paramount importance among studies of children and adolescents, who are frequently the main target groups of dental services and the main information source on dental needs assessments. We hypothesized that good oral habits and a healthy diet should be associated with higher levels of OHRQoL, as a consequence of the better oral health. Moreover it would be interesting to know and quantify the effect of certain foods on dental status and on OHRQoL to make specific impact-related strategies when setting priorities in prevention programs.

Portugal has a high DMFT index (decayed, missed and filled teeth) compared to other European countries (10). Although its association with diet has been evidenced, little is known about the relationship of diet and OHRQoL in adolescents. The objectives of this study were to assess the impact of diet and behavioural risk factors on caries and on OHRQoL among Portuguese adolescents.

## Material and Methods

A cluster-based random sample was carried out in 8 schools in Viseu (central Portugal), with a total of 16 schools from the 3rd cycle of basic education (3º CEB). This research was conducted according to the Helsinki Declaration. The methodology was authorized by the Ethics Committee of the School of Health of Viseu and it has been described in detail elsewhere by Bica *et al.* (11) Briefly, 1115 adolescents (between 11-17 yrs) were asked for self-perceived general status health, about brushing habits and about the using of toothpaste with fluoride. Moreover a Food Frequency Questionnaire was applied to quantify the frequency of consumption (never, less once a week, once a week, twice or three times a week, once a day, more than once a day) of the following groups of foods: cariostatic food (milk, yogurt/cheese, vegetables/salad, meet, fish, eggs, fresh fruit, nuts, sugarless gum) and cariogenic food (soup, bread, butter, bread with chocolate, sweetened popcorn, chocolate flakes, rice, pasta, baked potatoes, fried potatoes, fast food, croquettes, canned food, mik with chocolate, milk with sugar, sweetened desserts, pastry, biscuits, chocolate tablet, candies, compotes/jams, sugar, honey, refreshments, juices, coffee, tea with sugar), following the classification of American Dietetic Association (ADA) (12) and Palmer (13).

A clinical oral examination was carried out according to WHO guidelines (14) to record the DMFT index. The plaque index (PI) was obtained using the developer plate (erythrosine solution of 2%) and examined the six predefined teeth, according to the criteria classification of Greene & Vermillion and DGS (15).

To evaluate the OHRQoL, the 49-items Oral Health Impact Profile questionnaire (OHIP-49) (16) was applied. It includes 49 items whose scores can range from 0 to 4 (never, hardly ever, occasionally, fairly often and very often). Sum of these items compose 7 domains: functional limitation (sum of items 1 to 7), physical pain (sum of items 8 to 14), psychological discomfort (sum of items 15 to 21), physical disability (sum of items 22 to 28), psychological disability (sum of items 29 to 36), social disability (sum of items 37 to 43), and handicap (sum of items 44 to 49). The simple scoring method (OHIP-SC) was used to calculate the prevalence of impacts of the adolescents for a certain threshold (frequency ≥ 2). This method calculates the number of items reported as “occasionally” or more frequently. This quantitative score was calculated for both the domain and total score. A higher score indicates lower levels of OHRQoL.

The processing and analysis of data was performed using the Statistical Package for Social Sciences (SPSS) version 20.0. Techniques of descriptive statistics (frequency distributions and statistical measures) and inferential statistical techniques (Pearson correlation coefficient) were used in this study. A multivariate model (linear regression model) was built to analyze the factors associated with DMFT and with the OHRQoL, integrating all possible confounding variables and using step-wise method.

## Results

Valid data for this study were given by 782 subjects (response rate: 70.1%).  Table 1 shows the socio-demographic, habits and clinical variables description in adolescents. Males were the 44.5 % and over half were aged 12 to 14 years. Most part of them considered their health good or very good. With respect to habits, 93% brushed their teeth everyday but 36.2% did not use toothpaste with fluoride. The DMFT mean was 2.32±2.51 teeth, and almost 95% had between 4 and 6 index teeth with plaque.

**Table 1:**
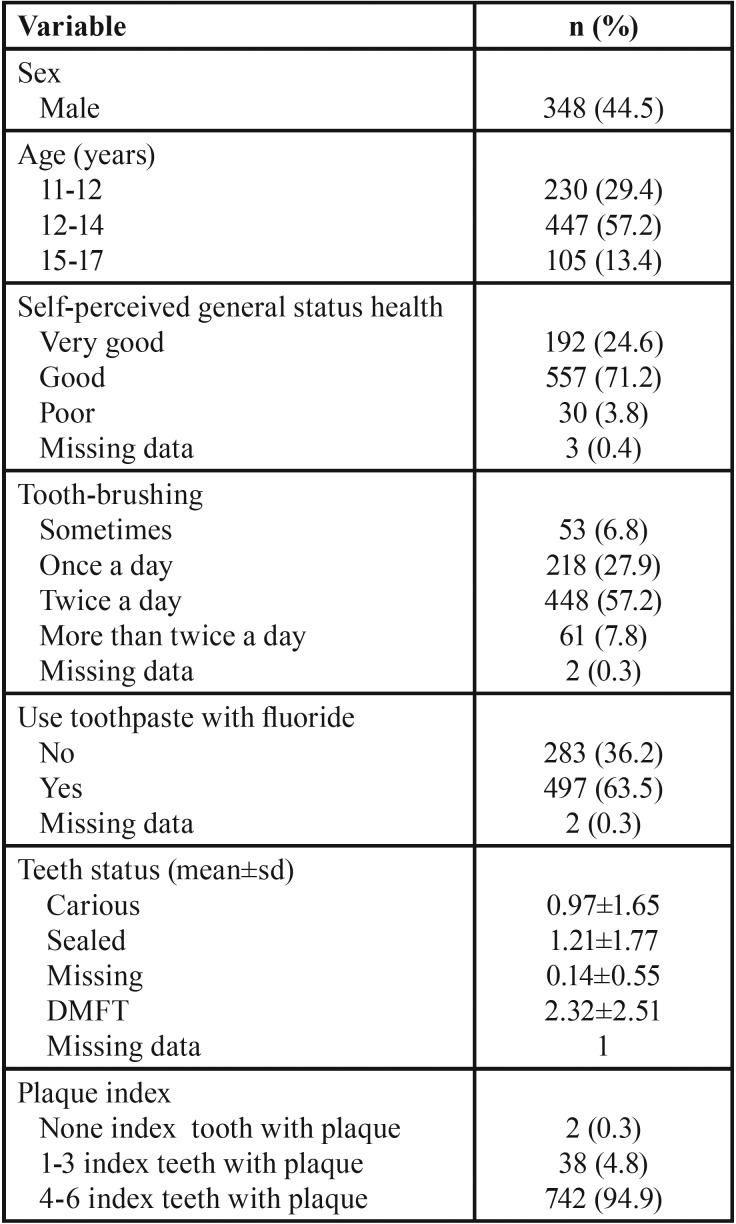
Description of sociodemographic, behavioral and clinical variables (n= 782).

The description of the OHRQoL appears in  Table 2. 56.3% of adolescents had at least one impact recorded as occasionally or most frequently. The most affected dimensions were functional limitation [mean±standard deviation (sd): 0.9±1.5 points] and physical pain (mean±sd: 0.8±1.4 points).

**Table 2:**
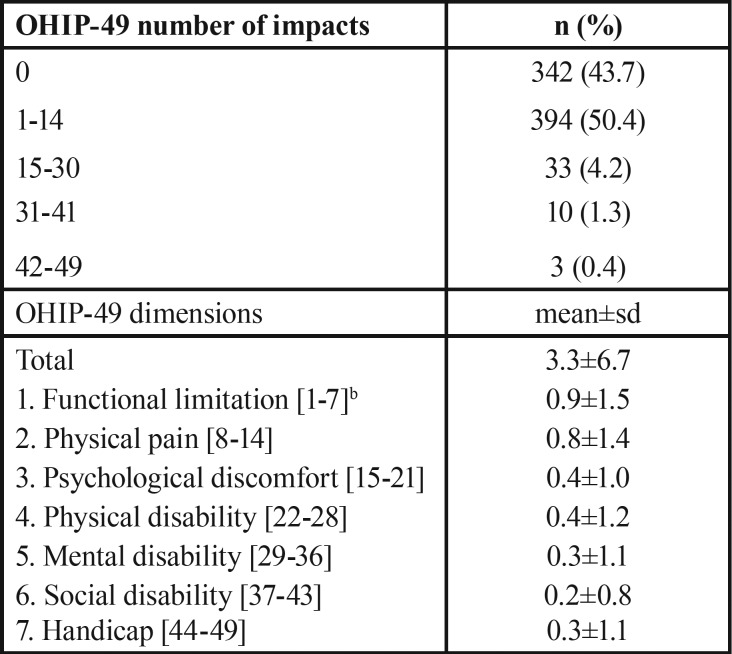
Description of oral health-related quality of life of adolescents: total and dimensions scores of OHIP-49 (simple count method)a (n= 782).

We analysed the frequency of consumption and the correlation between food consumption and both the DMFT index and the impact on OHRQoL ( Table 3). All the foods that were found to be significantly associated with either DMFT or OHRQoL were cariogenic. Chocolate consumption was common in the adolescent diet, through several formats, i.e. milk with chocolate (53.8%), biscuits (48.1%) and chocolate flakes (46.7%). DMFT was significantly associated with all of them, except chocolate flakes and fast food. By contrast, chocolate flakes were the food that impacted in more OHRQoL dimensions as well as in OHRQoL global. Psycological Disability and Social Disability were the dimensions more significantly correlated with this food consumption.

**Table 3:**
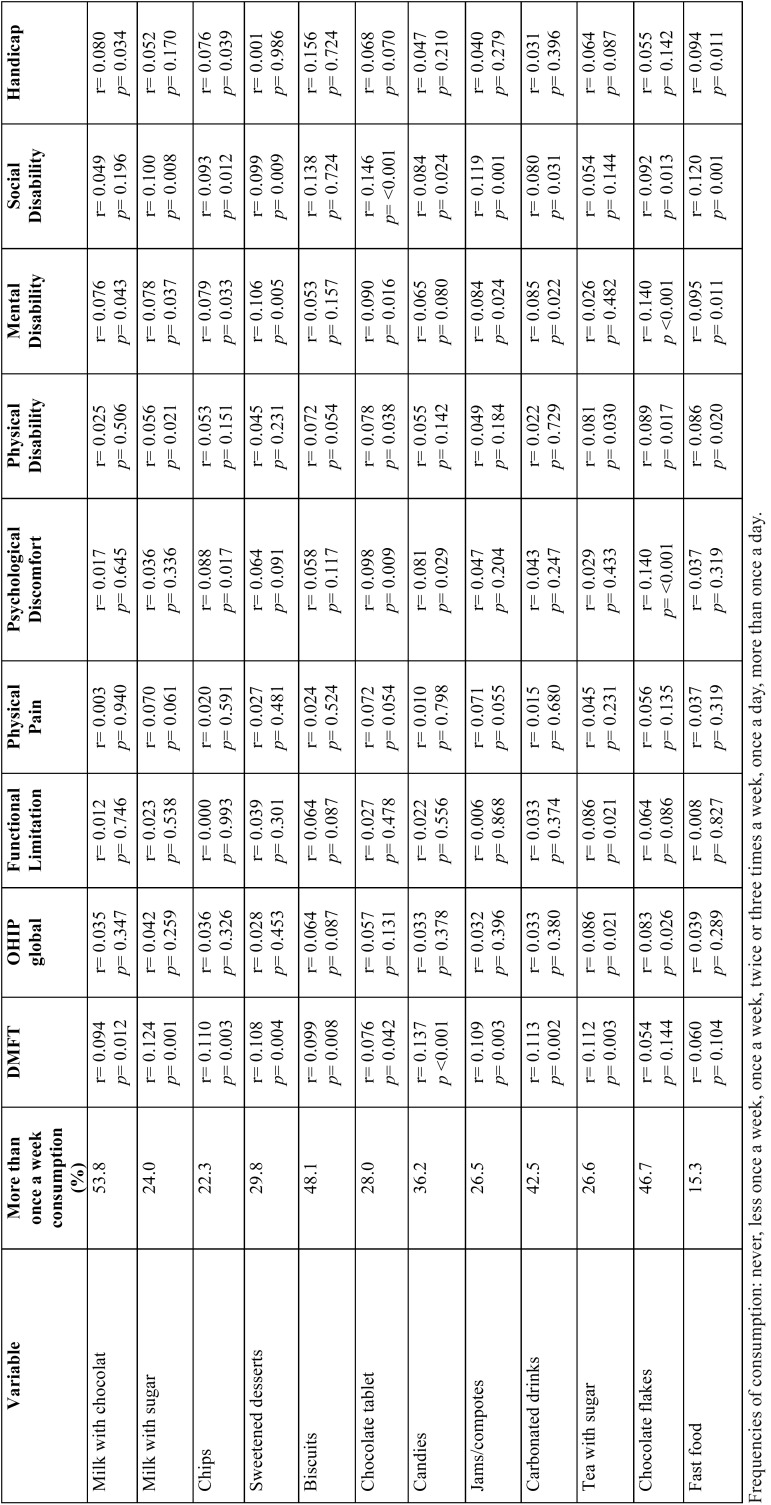
Description of frequency of food consumption a and Pearson correlations with oral health and oral health-quality of life (n=782).

A multivariate regression model for predicting the status of the caries and the impact on OHRQoL after including all the potential predictors (sociodemographic, behavioural and nutritional) is depicted in  Table 4. Age was significantly associated with both variables: the older the child the greater the DMFT index and the OHRQoL impact. Consumption more than once a week of tea with sugar, milk with sugar and biscuits remained in the model associated with DMFT. In addition to the lower levels of OHRQoL was reported by students who consumed frequently (more than once a week) fast food, chocolate flakes and those who brushed their teeth once a day or less frequently instead of 2-3 times a day.

**Table 4:**
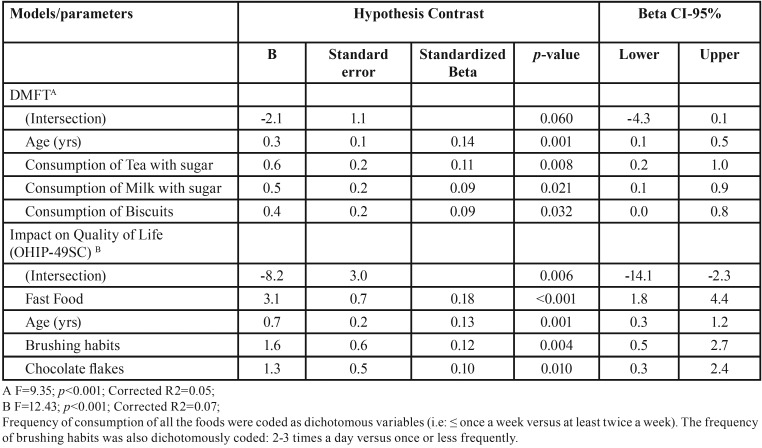
Step-wise linear regression model of DMFT and OHRQoL after including age, sex, brushing habits and the frequency of intake of all the food recorded.

## Discussion

This epidemiological study shows that the dietary habits influence on caries and on OHRQoL in Portuguese adolescents. It should be noted that this study has a series of limitations that should be borne in mind before proceeding to discuss the most relevant results. Although it is appropriated to the proposed objectives, the cross-sectional design of the study does not allow cause-effect relationships to be established. On the other hand, there is an absence of information about the bias which could arise from non-response but the proportion of responding subjects (70.1%) is acceptable and the data available were useful to achieve the objective of this study. Another potential limitation is the use of WHO criteria in the diagnosis of caries. This caries diagnostic criteria is based on the decay in dentin, which could be a limitation in not diagnosing the caries in their initial state or limited to enamel, mainly within these ages. Moreover, using a closed questionnaire in the evaluation of the dietary intake may have implied a partial loss of information about food consumption. Last, we selected a general OHRQoL measure (OHIP-49 questionnaire) which could difficult the comparison with other studies. It was chosen taking into account that the translation, cultural adaptation and validation into Brazilian Portuguese proved to be a reproducible and valid parameter for evaluating the impact of oral conditions on the quality of life of Brazilians. It was useful to reach our objective.

Society lifestyles are the most important determinant of both the general and oral health status (17). The association between caries and specific life styles, such as the consumption of cariogenic foods, the frequency of brushing, the frequency of check-ups and the socio-economic level, is well documented (18). 65.1 % brushed their teeth twice or more times a day. This result is better to those found in other previous studies whose prevalence of brushing was 55.6% twice a day or between 45% and 65% at least once a day (19-21). However, 36.2% of our sample used toothpaste without fluoride. Although benefits of topical fluorides are firmly established (22), it may be low awareness between adolescents and their parents as it has been described in previous studies (23). This result evidences that the state of knowledge concerning tooth-brushing and fluoride toothpaste needs to be improved.

The mean value of DMFT index was high (2.32) and it is a long way from the 1.50 recommended by WHO for 12 years-old children for 2020 (24). We have found that consumption more than once a week of sweetened food such as milk with chocolate, sweetened desserts, candies, chocolate tablets, jams/compotes, carbonated drinks, milk with sugar and biscuits were significantly associated with higher DMFT keeping the last two in the multivariate model. These results are consistent with the demonstrated role of sugar as a risk factor in the initiation and progression of dental caries (25). Consumption of tea with sugar also was significantly related to higher DMFT in the adjusted model. In spite of tea being a drink which seems to have preventive properties against dental caries (26), if it is consumed frequently and with too much sugar, it can become cariogenic agent.

We have integrated OHRQoL measure in this study in line with current trends stating the importance of integrating OHRQoL when assessing adolescents’s oral health status or needs. The range of presence of any oral impact in adolescents is very wide (from 28.6% to 94.5%) (27). In our study, an oral impact occasionally or more frequently was reported by more than half of patients (56.3%). The most affected dimension, functional limitation, is in relation to speaking and eating problems. This result is similar to other previous studies done in this population where eating was the most commonly affected performance (28,29). Due to demonstrated evidence of the inseparable relationship between perceived oral health and general health, this high prevalence of oral impacts occurrence in adolescents is an aspect for evaluation and monitoring. To best understanding, we also consider relevant to investigate the modulating factors of OHRQoL. As reported elsewhere (30), brushing habits were found to be significantly associated with OHRQoL. Consumption of fast food and chocolate flakes more than once a week also were associated with lower levels of OHRQoL.

Although more studies are needed to confirm the present results, the knowledge of underlying factors should allow suitable interventions to be developed for modifying some population-related nutritional habits, based on the observed risks for caries and OHRQoL. School educational programmes and the monitoring of these aspects could be beneficial.

In conclusion, frequency of consumption of sweetened/fast food was an important factor associated with caries and with OHRQoL.
